# Anesthetic Management for the Excision of a Pedunculated Vallecular Cyst Causing Airway Obstruction: A Case Report

**DOI:** 10.7759/cureus.83018

**Published:** 2025-04-25

**Authors:** Thandassery J Kannan, Asish Karthik

**Affiliations:** 1 Department of Anesthesiology and Critical Care, Government Medical College, Thrissur, Thrissur, IND

**Keywords:** airway management, airway obstruction, anesthesia, aspiration pneumonia, bronchoscopy, conscious sedation

## Abstract

Anesthetic management of vallecular cysts presents unique challenges, and available literature on the subject remains limited and varied. A 41-year-old female presented with dysphagia and positional dyspnea. Evaluation using video laryngoscopy revealed a 4 × 4 cm pedunculated cyst on the left side of the vallecula. The cyst was mobile, occasionally crossing the midline, and extended to the laryngeal surface, obscuring the laryngeal inlet. Visualization of the bilateral vocal cords was difficult and only possible with extensive maneuvering around the cyst. The cyst was scheduled for excision under general anesthesia, with anticipated challenges including abnormal positioning and potential airway collapse during induction. Awake fiberoptic intubation with a reinforced tube, performed in the lateral position, provided the safest approach. This case highlights that fiberoptic intubation with carefully titrated airway anesthesia and sedation, performed in the lateral position, can be a successful strategy in managing vallecular cysts.

## Introduction

A vallecular cyst, also referred to as a ductal cyst or mucus retention cyst, forms due to the accumulation of mucus secondary to obstruction of the collecting duct in a submucosal gland. Its exact incidence is unknown owing to its rarity. These cysts are predominantly reported in the pediatric population and are often asymptomatic; however, they can occasionally present as upper airway obstruction. When surgical excision is planned as definitive treatment, major anesthetic concerns include a difficult airway, risk of airway obstruction, cyst rupture, and potential pulmonary aspiration [1]. Various airway management techniques have been described, such as pre-intubation cyst aspiration to reduce its size, rigid bronchoscopy, and primary tracheostomy [1-3]. In our case, we employed flexible bronchoscopy as the primary airway management strategy for this unique presentation of a vallecular cyst, which, to our knowledge, is the first of its kind reported in the literature to date.

## Case presentation

**Figure 1:**
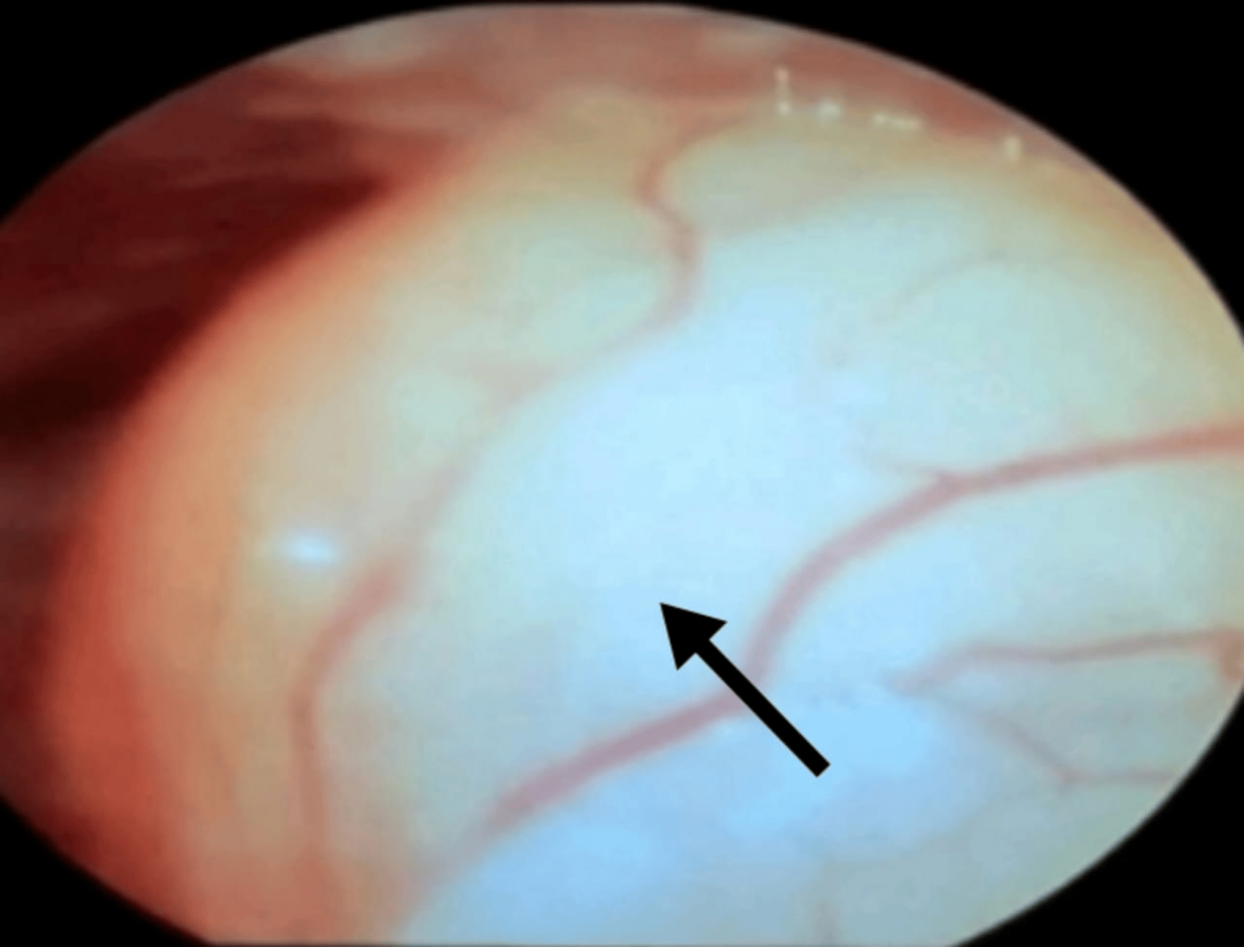
Videolaryngoscopic view showing the vallecular cyst (indicated by black arrow)

**Figure 2:**
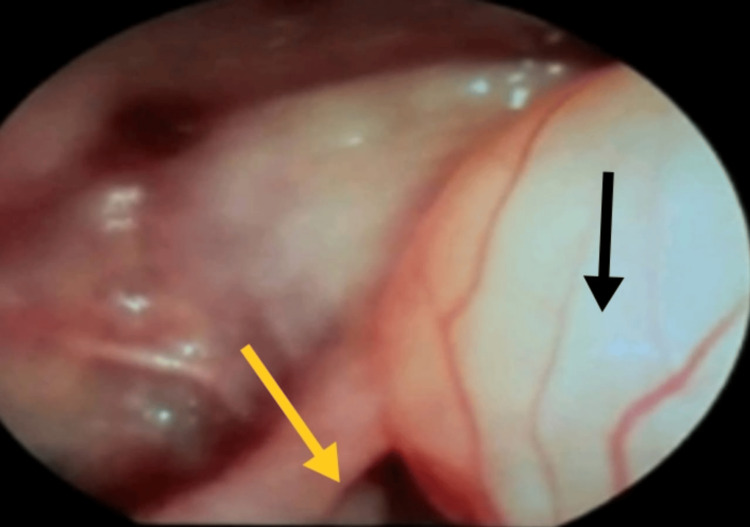
Videolaryngoscopic view showing the vallecular cyst (black arrow) nearly obstructing the laryngeal inlet (yellow arrow) The laryngeal inlet is visualized with significant difficulty.

**Figure 3:**
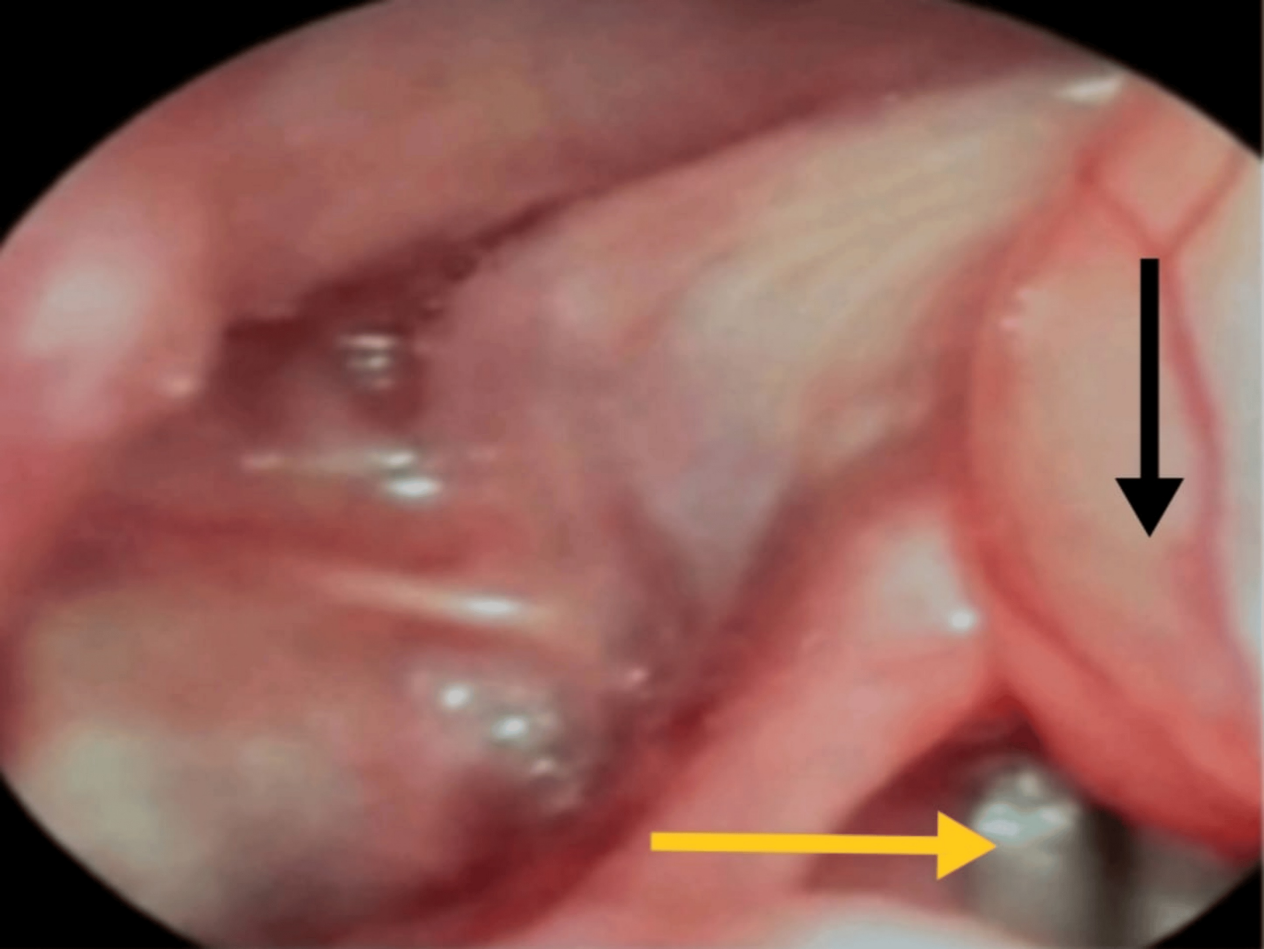
Videolaryngoscopic view showing the vocal cords (yellow arrow), visualized with difficulty through careful maneuvering around the cyst (black arrow) In this particular patient, the pedunculated and mobile nature of the pathology required very careful maneuvering around the lesion, with adjustments to the viewing angle and instrumentation made as needed.

A 41-year-old woman presented with gradually progressive dyspnea and dysphagia, along with exertional breathlessness over the past seven months. By the time of presentation, she could only lie flat in the left lateral position, which affected her sleep and activities of daily living. In the supine position, she experienced hoarseness and dyspnea. She had no significant comorbidities, and her investigations were within normal limits. On examination, she had a short and thick neck, with her airway assessed as modified Mallampati class II, normal thyromental distance, and normal temporomandibular joint mobility. The cyst was visible as a mass upon protrusion of the tongue. Video laryngoscopy revealed a 4 × 4 cm pedunculated, mobile cyst over the left vallecula, crossing the midline and extending to the laryngeal surface (Figure 1, Figure 2), which caused dyspnea in the supine position. The vocal cords were visualized only with difficulty, requiring careful maneuvering around the cyst (Figure 3). Bilateral vocal cords appeared normal and mobile. The patient also had grade II tonsillar hypertrophy.

Given the unstable and potentially collapsible airway, we planned an awake fiberoptic intubation, which was thoroughly explained to the patient to ensure optimal cooperation and alleviate her anxiety. She received glycopyrrolate 0.2 mg IV, dexamethasone 8 mg IV, midazolam 0.5 mg IV, and fentanyl 25 mcg IV. A Dexmedetomidine infusion was initiated, and she had received pantoprazole 40 mg PO and metoclopramide 10 mg as oral premedication the previous night. She was kept nil per os for more than eight hours, and her basal vitals, recorded after attaching the standard American Society of Anesthesiologists monitors, were as follows: HR 92/min, NIBP 128/90 mmHg, SpO2 98% RA, and RR 16/min.

To initiate topical anesthesia, she gargled 10 ml of 4% lignocaine viscous solution for five minutes, followed by nebulization with 4 ml of 4% lignocaine and 10% lignocaine spray in the oral cavity. However, unexpectedly, she developed dyspnea during this process and assumed a lateral recumbent position. Airway anesthesia was continued with nebulization of 2% lignocaine with adrenaline in the same recumbent position, augmented with 10% lignocaine spray. Oxygenation was managed with high-flow oxygen (6-8 L) via a nasal catheter, with its tip positioned at the nasopharyngeal inlet.

**Figure 4:**
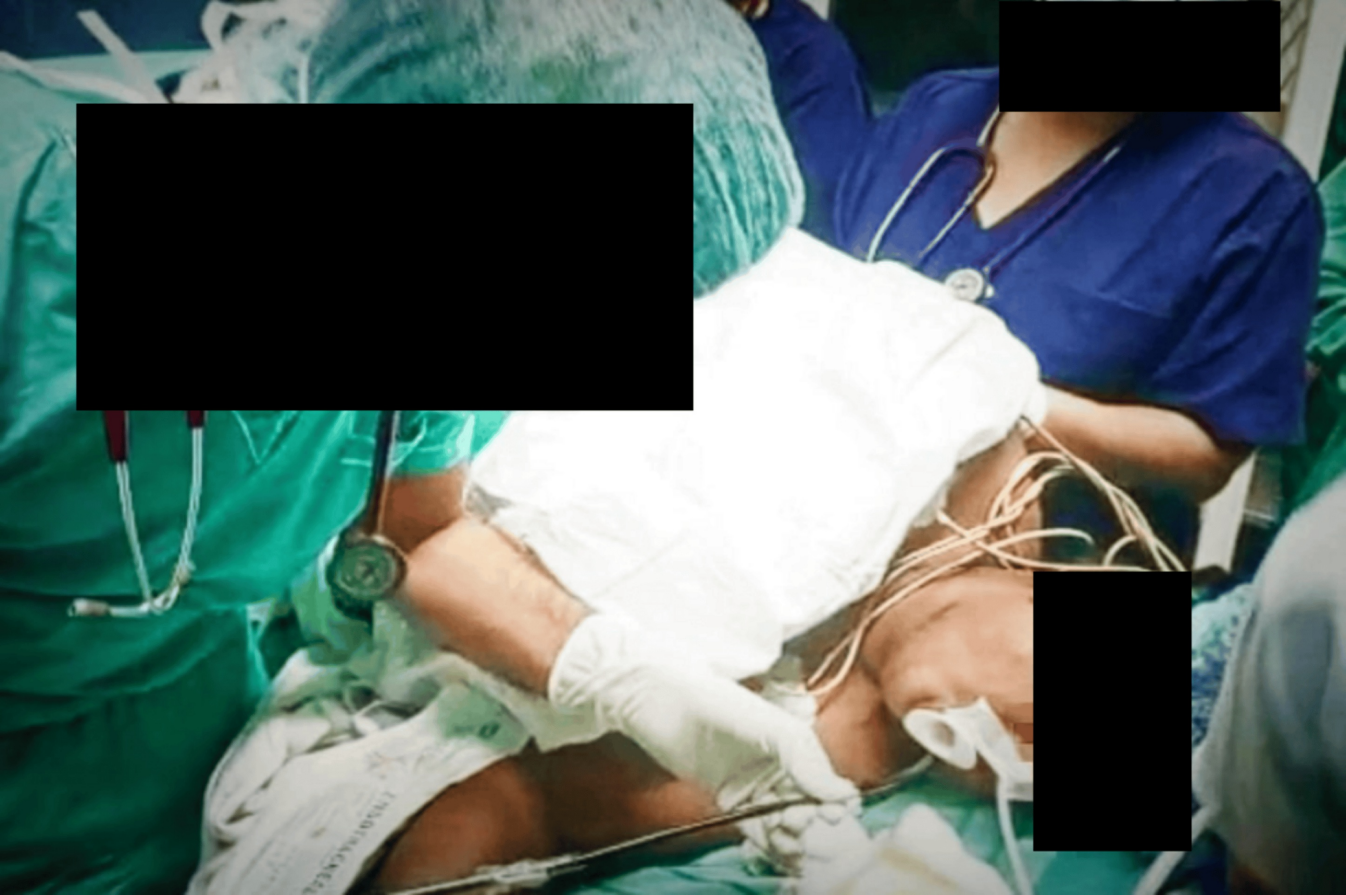
Fiberoptic bronchoscopy performed with the patient in the lateral decubitus position

A fiberoptic bronchoscope (FOB) with a loaded flexometallic endotracheal tube (size 7.0) was passed through the bite block placed between her incisors (Figure 4). The cyst was visualized, the diagnosis confirmed, and the FOB was carefully maneuvered around the cyst to visualize the glottic opening. Precautions were taken to avoid injury to the cyst, as any movement could rupture it. Patient sedation and comfort were optimized with bolus doses of fentanyl (25 + 25 mcg), propofol (30 + 30 mg), and midazolam (0.5 + 0.5 mg), all administered alongside the dexmedetomidine infusion. The FOB was maneuvered down to the carina, and the tube was threaded into the optimal position, 3 cm above the carina.

The plane of anesthesia and sedation was deepened with 60 mg of propofol IV, and a skeletal muscle relaxant, vecuronium 5 mg IV, was administered. Anesthesia was maintained with an air-oxygen-isoflurane (0.2-1%) mixture, titrated according to hemodynamic response. Surgery proceeded with the patient’s vital signs continuously monitored and stable throughout the procedure. The vallecular cyst was excised uneventfully.

Post-surgery, the neuromuscular blockade was reversed with 2.5 mg of neostigmine and 0.5 mg of glycopyrrolate IV, and the patient was extubated without complications. The patient had an uneventful recovery and was discharged on postoperative day 5 with complete resolution of symptoms.

## Discussion

Anesthetic management of a vallecular cyst involves addressing a difficult airway, the risk of cyst rupture, and potential aspiration. In this patient, the cyst was large, pedunculated, and mobile. The glottic opening was not visible during indirect laryngoscopy and was only visualized with significant difficulty during video laryngoscopy, where only the lateral margins were visible. This necessitated the use of an FOB, which also helped rule out other differential diagnoses, such as lingual tonsil hypertrophy and epiglottic cysts, highlighting its diagnostic utility.

However, the patient was dyspneic in all positions except the left lateral position, creating a challenge in positioning. The use of FOB in the lateral position is difficult because the visuospatial orientation is altered. Anesthesiologists are typically trained to use the FOB with the patient in the supine or sitting position. In this case, being in the lateral position required modifications to the conventional planes of FOB manipulation, which posed practical difficulties due to the altered orientation.

Additionally, optimal airway anesthesia was crucial for patient compliance, as any movement during FOB manipulation could lead to cyst rupture, aspiration, or laryngospasm, especially when the protective reflexes were diminished. Preventing rupture was essential because the exact nature of the cyst was unknown, and rupture or aspiration of the contents of an infected cyst posed increased risks. The risk of rupture was present throughout the procedure, including during FOB manipulation, railroading of the endotracheal tube, and patient movement.

In the event of rupture, the contingency plan involved positioning the patient in Trendelenburg, with quick suction and intubation via direct laryngoscopy. Additionally, airway nerve blocks were not feasible due to the distorted anatomy, so airway anesthesia was achieved with lignocaine nebulization, gargle, and oral spray.

The literature on anesthetic management of adult vallecular cysts is scarce and anecdotal. Although typically asymptomatic, vallecular cysts can lead to fatal complications. No specific management technique has been described. In neonates, para-glossal laryngoscopy has been used successfully, and inhalational induction followed by intubation after muscle relaxation has also been described; however, there is a risk of airway obstruction, especially in cases of associated laryngomalacia. Cyst aspiration before intubation may lead to inadvertent pulmonary aspiration, recurrence, and difficulty identifying the cyst margins during future surgical excisions [4]. A rigid laryngoscope may also be employed, as it can displace the cyst to one side, providing a reliable backup option. Concurrent planning for a definitive airway is essential due to the high risk of airway loss, especially in cases involving infected cysts, which may lead to rapid airway obstruction due to acute epiglottitis or abscess formation [5].

Awake fiberoptic bronchoscopy is considered the safest option, but it requires meticulous planning given the technical difficulty, fragile nature of the cyst, and distorted anatomy. A contingency plan is necessary in case of failure. Although fiberoptic intubation in the lateral position is uncommon, it was required in this case for patient comfort. With carefully tailored airway anesthesia and sedation, FOB was performed in the lateral position without complications. A definitive surgical airway, such as tracheostomy, should always be planned as a lifesaving backup, in consultation with the ENT surgeon. Airway loss during induction and multiple failed intubation attempts leading to hypoxia and bradycardia have been described as a scenario necessitating tracheostomy [6].

Although the nasal route is commonly used, we selected the oral route for FOB introduction, as it is easier to manipulate in emergency situations such as cyst rupture or airway obstruction. Additionally, our patient had a deviated nasal septum and unevaluated turbinate hypertrophy.

This report presents a single case of successful management of a rare condition with a unique presentation. The management approach used in this case can be adapted to individual patients, considering the rarity of the pathology, clinical presentation, and patient safety.

## Conclusions

This patient presented a unique challenge of an unstable airway. Among the various options available, fiberoptic intubation emerged as the best choice. When performed with carefully titrated airway anesthesia and sedation in the lateral position, fiberoptic intubation can be successfully employed in the management of vallecular cysts.
